# Effect of Pravastatin as an Adjunctive Therapeutic for Mitral Insufficiency with Hyperlipidemia in a Dog

**DOI:** 10.1155/2021/6054125

**Published:** 2021-09-03

**Authors:** Yu Sahashi, Miwako Sahashi, Yoshiaki Hikasa

**Affiliations:** ^1^Sahashi Veterinary Hospital, 23−10 Kitabatake, Ueno, Inagawa, Kawabe-gun, Hyogo 666-0244, Japan; ^2^Laboratory of Veterinary Internal Medicine, Department of Clinical Veterinary Science, Joint Graduate School of Veterinary Sciences, Tottori University, 4-101 Koyama Minami, Tottori 680-8553, Japan

## Abstract

Pravastatin (PS) has been found to increase left ventricle (LV) expansion capacity and decrease LV constriction and left atrial pressure in healthy dogs. To date, there are no available reports on the effects of PS in dogs with hypercholesterolemia with chronic heart failure (CHF). This case report demonstrates a successful long-term treatment plan using PS in a dog suffering from mitral insufficiency with hyperlipidemia. A 12-year-old, castrated male Chihuahua dog had mitral insufficiency with hyperlipidemia. The dog presented with symptoms of chronic coughing. PS was orally administered (1 mg/kg, SID) in addition to general treatment for mitral insufficiency. The follow-up period was 375 days. PS administration decreased the heart rate (HR), vertebral heart size (VHS), and N-terminal probrain natriuretic peptide (NT-proBNP) concentration of the dog. In addition, PS administration also improved chronic cardiac failure induced by mitral insufficiency and hyperlipidemia. This report suggests that PS can be useful as an adjunctive therapeutic for dogs with hypercholesterolemia with mitral insufficiency.

## 1. Introduction

Statins are 3-hydroxy-3-methyl-glutaryl-CoA (HMG-CoA) reductase inhibitors that reduce blood cholesterol levels by inhibiting the HMG-CoA reductase in the mevalonic acid pathway [1]. In addition, this group of medications has been shown to have multifaceted effects, including antimyocardial hypertrophy, anti-inflammatory effects, and antioxidant activity [1]. Statins activate the P13 kinase-Akt pathway by inhibiting the mevalonate pathway. Statins also activate endothelial nitric oxide synthase (eNOS), followed by an increase in nitric oxide (NO) production [1, 2]. Pravastatin (PS), a statin medication, has been reported to prevent cardiomyocyte cell death via an increase in NO release, a decrease in myocyte endothelin-1 production, and an increase in protein kinase Akt activation [3]. Moreover, statins have been described to modulate both the neurohormonal activation and the autonomic nervous system by activating eNOS and increasing NO production [1]. For example, it has been shown that elevated cholesterol levels are associated with the overexpression of angiotensin II type 1 receptors [4] and that statins decrease the levels of these receptors, thus resulting in both decreased angiotensin II-mediated vasoconstriction and enhanced response to angiotensin receptor blockers [5–7]. Furthermore, simvastatin has been reported to normalize sympathetic outflow and reflex regulation in rabbits with chronic heart failure (CHF) [8]. Therefore, these drugs may be useful for the treatment of CHF with hypercholesterolemia in dogs. In a previous study, we have reported that PS increases left ventricle (LV) expansion capacity and decreases LV constriction and left atrial pressure in healthy dogs [9]. Consequently, we suggested that PS may be effective in improving heart failure with LV diastolic dysfunction or elevated left atrial pressure in dogs. To date, no reports are available on the effects of PS in dogs with hypercholesterolemia with CHF. This case report demonstrates a successful long-term treatment plan using PS in a dog suffering from mitral insufficiency with hyperlipidemia.

## 2. Case Presentation

A 12-year-old, castrated male Chihuahua dog weighing 4.2 kg was referred to the hospital with a diagnosis of chronic coughing. The medical history of the dog included tracheal collapse. Physical examinations revealed a grade III murmur and cough. Respiration rate was 20 breaths/min. The dog was diagnosed with hyperlipidemia and mitral insufficiency whose severity was classified as II b by the International Small Animal Cardiac Health Council. Consequently, the administration of alacepril (1.5 mg/kg, SID) and a low-fat meal were initially prescribed. The subsequent treatment was continued for a period of two months. However, cardiomegaly continued to progress, whereas the frequency of coughing was not reduced. Moreover, hyperlipidemia was not improved. Abdominal ultrasonography revealed biliary debris that was gradually increasing in size. Consequently, PS (1 mg/kg, SID) was prescribed. Following PS administration, the clinical signs were evaluated, and hematologic, serum biochemical, and radiologic examinations and echocardiographic and circulation parameter measurements were performed.

Cough disappeared one week after PS administration. In addition, blood biochemical tests revealed that the total cholesterol, triglyceride, and N-terminal probrain natriuretic peptide (NT-proBNP) concentrations were significantly decreased from Day 11 to Day 211 (Table 1). An increase in creatine kinase value was not observed throughout the administration period. Additional hematologic and biochemical findings indicating renal or hepatic failures and adverse effects were not observed up to 375 days after PS administration (Table 1).

Furthermore, the radiographic vertebral heart size (VHS) decreased after PS administration, as shown in Table 2. With respect to the echocardiographic and circulation variables, decreases in heart rate (HR) and left atrium/aorta ratio (LA/Ao) were observed during PS administration, whereas LV fractional shortening (FS) and LV ejection fraction (EF) increased from Day 30 to Day 150 after PS administration (Table 2). No marked changes in the peak velocity of early diastolic transmitral flow (*E*), peak velocity of late transmitral flow (*A*), *E*/*A* ratio, and deceleration time of early diastolic transmitral flow (DT_E_) were observed during PS administration. Stroke volume (SV) and cardiac output (CO) decreased on Days 30 and 45 after PS administration but thereafter increased and maintained well. No substantial changes in the maximum systolic mitral regurgitation velocity (MRmax) were identified after PS administration. The maximum systolic tricuspid regurgitation velocity (TRmax) and systolic pulmonary arterial pressure (sPA), calculated using the modified Bernoulli equation (4 x TRmax^2^ + 10), both decreased after PS administration (Table 2). The peak velocity of early diastolic mitral annular motion (Em)/peak velocity of diastolic mitral annular motion (Am) ratio increased during PS administration compared with preadministration. *E*/Em decreased on Days 34 and 65 after PS administration but increased after Day 96, and a higher value was then observed (Table 2).

## 3. Discussion

In dogs with mitral insufficiency, increased HR, LA/Ao ratio and serum NT-proBNP concentration, and pulmonary hypertension (PH) can be considered negative prognostic factors [10, 11]. However, our case study reveals that HR apparently decreased after PS administration. This may be due to the PS-induced inhibition of the sympathetic nerve activity via blocking angiotensin II-mediated activity [1]. In addition, VHS, LA/Ao ratio, sPA, and serum NT-proBNP concentration decreased after PS administration, which could be improved with the prognosis.

Furthermore, we identified that both SV and CO decreased on Days 30 and 45 after PS administration but thereafter increased. In fact, SV reached a higher value compared with before PS administration. As statins activate eNOS followed by an increase in NO production and inhibit the Rho GTPases resulting in peripheral arterial vasodilatation [1, 2], we speculated that the increase in CO may be due to the improved heart failure facilitated by cardiac afterload reduction after PS administration. In this case, however, CO did not apparently increase after PS administration, which could be attributed to the decrease in HR after PS administration.

Furthermore, MRmax did not markedly change from the baseline value. In contrast, both TRmax and sPA decreased after PS administration, which may be caused by the pulmonary artery dilation from the inhibitory effect of statins on Rho kinase [12]. In addition, because the decreased eNOS expression [12] and increased endothelin-1 expression [13] have been identified as causes of PH, both PS-induced eNOS activation [2] and decreased ET-1 expression [3] may be involved in sPA reduction. *E*/Em temporarily decreased after PS administration, thus suggesting that PS administration decreases left atrial pressure reduction. Finally, the decrease in serum NT-proBNP observed after the administration of PS may be associated with the reduction of cardiac load facilitated by the stain-induced inhibition of angiotensin II type 1 receptor expression and vascular contraction by angiotensin II [1].

In this case, coughing was apparently improved after PS administration. This may be due to cardiac load reduction as evidenced by the decrease in VHS and LA/Ao ratio after PS administration. In contrast, statins are known to have anti-inflammatory effects by acting on several pathways or factors, such as cholesterol biosynthesis pathway, Ras and Rho kinase, nuclear factor-*κ*B and activator protein-1-mediated proinflammatory pathways, and peroxisome proliferator-activated receptor and Kruppel-like factor-2 [14]. Therefore, coughing improvement following PS administration may be due to the multifaceted effects of statins, including cardiac load reduction and anti-inflammatory effects.

In conclusion, PS administration in addition to the general treatment for mitral insufficiency with hyperlipidemia in a dog was found to decrease tachycardia and left atrial pressure and also improved hyperlipidemia and PH without reducing blood pressure. Moreover, the clinical symptoms of chronic cardiac failure were also improved. Therefore, PS administration may be effective as an adjunctive therapy in improving cardiac function and lipid metabolism in dogs with both mitral insufficiency and hyperlipidemia.

## Figures and Tables

**Table 1:** Hematological, serum biochemical, and cardiac biomarker parameters after pravastatin administration.

Variables	Reference	Time (days)										
	Range	0	11	30	45	65	96	121	150	211	283	375
White blood cells (×10^3^/mm^3^)	6 − 17	12.0	—	10.1	—	11.1	10.7	8.7	9.1	10.6	9.4	11.2
Red blood cells (×10^4^/mm^3^)	550−850	652	—	656	—	723	758	645	718	676	695	686
Packed cell volume (%)	37−55	45.1	—	45.1	—	49.9	51.6	44.3	49.9	46.4	47.3	47.0
Alanine aminotransferase (U/L)	10−100	59	—	102	—	109	112	118	123	105	92	98
Aspartate aminotransferase (U/L)	0 − 50	22	—	25	—	27	28	24	30	29	28	30
Alkaline phosphatase (U/L)	23−212	443	—	297	—	319	361	330	367	303	244	242
Blood urea nitrogen (mg/dL)	7 − 27	14.3	—	16.3	—	17.2	13.4	17.7	19.9	17.2	14.4	18.2
Creatinine (mg/dL)	0.5 − 1.8	0.6	—	0.8	—	0.8	0.8	0.6	0.6	0.7	0.8	0.7
Triglyceride (mg/dL)	17−79	577	396	180	178	424	402	209	205	263	162	294
Total cholesterol (mg/dL)	111−320	360	281	274	273	279	295	295	317	256	253	239
Creatine kinase (U/L)	10−200	60	75	69	75	98	75	63	70	79	75	166
Serum N-terminal probrain natriuretic peptide (pmol/L)	<900	2449	1352	1117	1402	1305	1209	1020	1058	988	2704	2146

**Table 2:** Echocardiographic and circulation variables after pravastatin administration.

Variables	Time (days)										
	0	11	30	45	65	96	121	150	211	283	375
Body weight (kg)	4.3	4.4	4.3	4.3	4.3	4.2	4.2	4.2	4.2	4.2	4.2
Radiographic vertebrae heart size (VHS)	11.0	10.5	10.3	10.5	10.3	10.0	10.0	10.5	10.0	9.8	10.5
Heart rate (beats/min)	150	139	132	107	132	130	119	110	101	114	131
Systolic blood pressure (SBP; mmHg)	126	168	154	148	148	160	178	164	156	153	154
Diastolic blood pressure (DBP; mmHg)	68	83	91	73	90	105	99	107	100	93	84
Mean blood pressure (MAP; mmHg)	87	100	109	100	105	135	120	135	121	122	104
Left atrium (LA; mm)	19.5	15.9	16.3	16.4	16.2	17.2	16.9	16.2	16.2	13.3	14.1
Right atrium (RA; mm)	13	12.3	12.3	12.5	11.5	11.9	13	11	13	10.2	11.4
Aorta (Ao; mm)	11.2	11.1	11.5	11.7	12.1	12.6	12.4	12.6	12.5	11.3	12.4
LA/Ao	1.74	1.43	1.42	1.40	1.34	1.37	1.36	1.29	1.29	1.18	1.14
RA/Ao	1.16	1.11	1.06	1.06	0.95	0.94	1.04	0.87	1.04	0.9	0.91
LV fractional shortening (FS; %)	57	43	52	47	47	51	43	47	45	43	43
LV ejection fraction (EF; %)	88	76	84	80	80	84	75	80	78	76	76
End-systolic left ventricular inner dimension (LVIDs; mm)	12.7	16.4	13	15.1	14	13.2	15.8	14.9	15.7	16.6	17
End-diastolic left ventricular inner dimension (LVIDd; mm)	29.6	28.7	26.8	28.7	26.3	26.8	27.4	28.1	29.6	29.4	30.1
Normalized end-diastolic left ventricular inner dimension (LVIDdN)	1.90	1.85	1.74	1.86	1.71	1.75	1.84	1.84	1.94	1.92	1.97
Pulmonary valve flow velocity (m/s)	0.78	0.78	1.00	0.89	1.09	1.01	0.89	1.03	0.80	0.82	0.89
Peak velocity of early diastolic transmitral flow wave (*E*; cm/s)	76.5	97.0	78.0	93.6	75.2	84.0	72.0	91.9	83.1	90.6	84.1
Peak velocity of late transmitral flow wave (*A*; cm/s)	94.3	70.2	62.3	66.3	71.1	58.7	61.5	61.7	69.6	60.4	65.7
*E*/*A*	0.81	1.38	1.25	1.41	1.06	1.43	1.17	1.49	1.19	1.5	1.28
Deceleration time of early diastolic transmitral flow (DTE; ms)	108	80	104	108	136	136	104	92	104	120	120
Stroke volume (SV; mL)	5.1	5.2	3.8	3.6	5.0	7.2	4.7	4.3	4.9	6.1	6.1
Aortic ejection flow velocity (AEV; m/s)	0.73	0.86	0.99	0.88	0.83	0.91	0.76	0.84	1.26	0.80	0.84
Cardiac output (CO; L/min)	0.668	0.680	0.381	0.339	0.544	0.694	0.484	0.633	0.494	0.522	0.497
Peak velocity of aortic valve regurgitation (AR; cm/s)	129	216	104	275	245	214	147	256	241	260	265
Maximum systolic mitral regurgitation velocity (MRmax; m/s)	5.76	6.10	5.98	5.86	5.32	5.63	5.40	5.64	5.70	5.25	5.54
Maximum tricuspid regurgitation velocity (TRmax; m/s)	3.20	2.97	2.17	2.73	2.88	2.83	2.63	2.67	2.67	2.60	2.23
Systolic pulmonary arterial pressure (sPA; mmHg)	51.0	45.3	28.8	39.8	43.2	42.0	37.7	38.5	38.5	37.0	29.9
Left ventricular Tei index	0.49	0.49	0.55	0.38	0.32	0.77	0.70	0.51	0.29	0.53	0.40
Right ventricular Tei index	0.13	0.30	0.18	0.22	0.37	0.40	0.23	0.33	0.30	0.66	0.55
Peak velocity of early diastolic mitral annular motion (Em; cm/s)	6.7	8.4	6.5	10.2	9.1	6.7	6.5	7.1	4.9	5.8	7.4
Peak velocity of diastolic mitral annular motion (Am; cm/s)	11.5	13.1	7.7	11.6	10.8	9.6	9.2	8.5	7.4	9.7	11
Em/Am	0.58	0.64	0.84	0.88	0.84	0.70	0.70	0.83	0.65	0.60	0.67
E/Em	11.5	11.5	12.1	9.2	8.2	12.6	11.0	12.9	16.9	16.1	9.5
Tricuspid annular plane systolic excursion (TAPSE; mm)	12.1	13.0	12.4	13.2	11.4	12.5	11.9	11.9	11.7	11.0	10.2

## Data Availability

The clinical data used to support the findings of this case are included within the text and in tables.

## Abbreviations

*A*: Peak velocity of late transmitral flow
Ao: Aorta
Am: Peak velocity of diastolic mitral annular motion
CHF: Chronic heart failure
CO: Cardiac output
DT_E_: Deceleration time of early diastolic transmitral flow
*E*: Peak velocity of early diastolic transmitral flow
EF: Ejection fraction
Em: Peak velocity of early diastolic mitral annular motion
eNOS: Endothelial nitric oxide synthase
FS: Fractional shortening
HMG-CoA: 3-Hydroxy-3-methyl-glutaryl-CoA
HR: Heart rate
LA: Left atrium
LV: Left ventricle
MRmax: Maximum systolic mitral regurgitation velocity
NO: Nitric oxide
NT-proBNP: N-terminal probrain natriuretic peptide
PH: Pulmonary hypertension
PS: Pravastatin
sPA: Systolic pulmonary arterial pressure
SV: Stroke volume
TRmax: Maximum systolic tricuspid regurgitation velocity
VHS: Vertebral heart size.
